# Universal Newborn Screening for Congenital Cytomegalovirus Infection – From Infant to Maternal Infection: A Prospective Multicenter Study

**DOI:** 10.3389/fped.2022.909646

**Published:** 2022-07-06

**Authors:** Angela Chiereghin, Claudia Pavia, Gabriele Turello, Eva Caterina Borgatti, Federico Baiesi Pillastrini, Liliana Gabrielli, Dino Gibertoni, Concetta Marsico, Massimo De Paschale, Maria Teresa Manco, Antonia Ruscitto, Laura Pogliani, Marta Bellini, Alessandro Porta, Luciana Parola, Maria Luisa Scarasciulli, Agata Calvario, Manuela Capozza, Maria Grazia Capretti, Nicola Laforgia, Pierangelo Clerici, Tiziana Lazzarotto

**Affiliations:** ^1^Microbiology Unit, IRCCS Azienda Ospedaliero-Universitaria di Bologna, Bologna, Italy; ^2^Microbiology Unit, ASST Ovest Milanese, Hospital of Legnano, Milan, Italy; ^3^Section of Microbiology, Department of Experimental, Diagnostic and Specialty Medicine, University of Bologna, Bologna, Italy; ^4^Research and Innovation Unit, IRCCS Azienda Ospedaliero-Universitaria di Bologna, Bologna, Italy; ^5^Neonatology Unit, IRCCS Azienda Ospedaliero-Universitaria di Bologna, Bologna, Italy; ^6^Pediatrics Unit, ASST Ovest Milanese, Hospital of Legnano, Milan, Italy; ^7^Pediatrics Unit, ASST Ovest Milanese, Hospital of Magenta, Milan, Italy; ^8^Microbiology and Virology Unit, Azienda Ospedaliero-Universitaria di Bari, Bari, Italy; ^9^Neonatology and NICU Unit, Interdisciplinary Department of Medicine, University of Bari, Bari, Italy

**Keywords:** universal newborn screening, congenital CMV infection, CMV maternal infection, salivary swabs, false positive results, levels of CMV-DNA

## Abstract

**Introduction:**

Most infants at risk for cytomegalovirus (CMV)-associated sensorineural hearing loss (SNHL) are unrecognized because of the absence of a universal neonatal CMV screening. The search of CMV-DNA by molecular methods in salivary swabs was demonstrated to be a reliable approach. This study describes the results obtained by carrying out a universal screening for congenital CMV (cCMV) infection including all live-born newborns in three Italian sites, as well as the therapeutic interventions and clinical outcome of the CMV-infected neonates. Moreover, CMV maternal infection's characteristics were evaluated.

**Methods:**

To confirm or exclude cCMV infection, a CMV-DNA-positive result on a first salivary swab was followed by repeated saliva and urine samples collected within 21 days of age. Breast milk samples were also collected. The search of CMV-DNA was performed with a single automated quantitative commercial real-time PCR assay, regardless of the type of samples used.

**Results:**

A total of 3,151 newborns were enrolled; 21 (0.66%) of them were congenitally infected (median saliva viral load at screening, 6.65 [range, 5.03–7.17] log_10_ IU/ml). Very low/low viral load in screening saliva samples (median value, 1.87 [range, 1.14–2.59] log_10_ IU/ml) was associated with false-positive results (*n* = 54; 1.7%). CMV-DNA was detected in almost half of the breast milk samples of mother–infant pairs with a false-positive result, suggesting that contamination from breast milk may not be the only explanation in the study population. cCMV infection confirmation with the search of CMV-DNA in a urine sample proved to be the gold standard strategy, since false-positive results were observed in 4/54 (7.5%) of the repeated saliva samples. Symptomatic cCMV infection was observed in 3/21 (14.3%) infants; notably, one (4.7%) developed moderate unilateral SNHL at 5 months after birth. Finally, two symptomatic cCMV infections were associated with primary maternal infection acquired in the first trimester of gestation; one newborn with severe cCMV symptoms was born to a mother with no CMV checkups in pregnancy.

**Conclusion:**

Without universal neonatal CMV screening, some infected infants who develop late neurological sequelae may not be recognized and, consequently, they are not able to benefit early from instrumental and therapeutic interventions to limit and/or treat CMV disease.

## Introduction

Congenital cytomegalovirus (cCMV) infection is a huge public health problem causing neurodevelopmental sequelae, including neurological disability and sensorineural hearing loss (SNHL) (1). The burden of CMV mother-to-child transmission is not completely realized since CMV-related clinical symptoms often do not manifest at birth (2). It has indeed been estimated that almost 10% of the asymptomatic congenitally CMV-infected neonates later develop hearing loss (3). Therefore, most infants at risk for CMV-associated SNHL are unrecognized because of the absence of a universal neonatal CMV screening (4) that could allow early detection of congenitally infected infants and consequently prompt interventions, improving the infants' clinical outcomes (5). Newborn screening for cCMV infection appeared to be cost-effective, as reported by Gantt and colleagues: they evaluated large prospective cohorts in the United States and reported that universal neonatal CMV screening generated larger net savings and the greatest opportunity to provide directed care (6). For large-scale universal neonatal cCMV screening, saliva samples obtained by buccal swabs seem to be an appropriate and non-invasive type of specimen to be analyzed by nucleic acid amplification test (NAAT), considering the high titers of CMV shed by congenitally infected newborns in the saliva and the easiness of specimen sampling (3, 7–10). However, a limitation of saliva samples is the possible contamination due to CMV-DNA present in the genital secretions in the birth canal or in milk from the last breastfeeding (11).

This prospective multicenter study aimed to assess the potential benefit of newborn screening for cCMV to early identify infected newborns and their clinical spectrum and analyze the association between neonatal CMV infection/disease and maternal CMV infection in pregnancy. Moreover, this study investigated if a viral DNA cutoff value in CMV-DNA-positive saliva samples collected within 21 days of life can discriminate a congenitally infected newborn from a non-congenitally infected newborn.

Finally, the study evaluated if a repeated saliva sample might replace the gold standard, represented by neonatal urine sample (11), as confirmatory testing for the diagnosis of cCMV infection.

## Methods

### Study Design

Neonatal cCMV screening was offered at birth to all the live newborns born in the period between 12 February 2019 and 21 July 2020 in 3 Italian sites (i.e., IRCCS Azienda Ospedaliero-Universitaria di Bologna, ASST Ovest Milanese, Hospitals of Legnano and Magenta [Milan], and Azienda Ospedaliero-Universitaria di Bari). The study population included newborns whose parents/guardians agreed to screen for cCMV infection and gave written consent for the inclusion in the study.

The sample size estimation carried out in the study design phase assessed that 20 positive subjects were needed to achieve 0.80 power for a one-sample proportion test in which *p0* = 0.50 was the null hypothesis of the no-discrimination curve and *p*1 = 0.80 was the expected area under the curve (AUC) of the molecular assay. After dividing this result by the expected cCMV prevalence in the population (0.64%) and accounting for an expected 1.3% of false-positive results (12–14), it was estimated that at least 3,125 subjects should have been recruited for this study.

**Figure 1:**
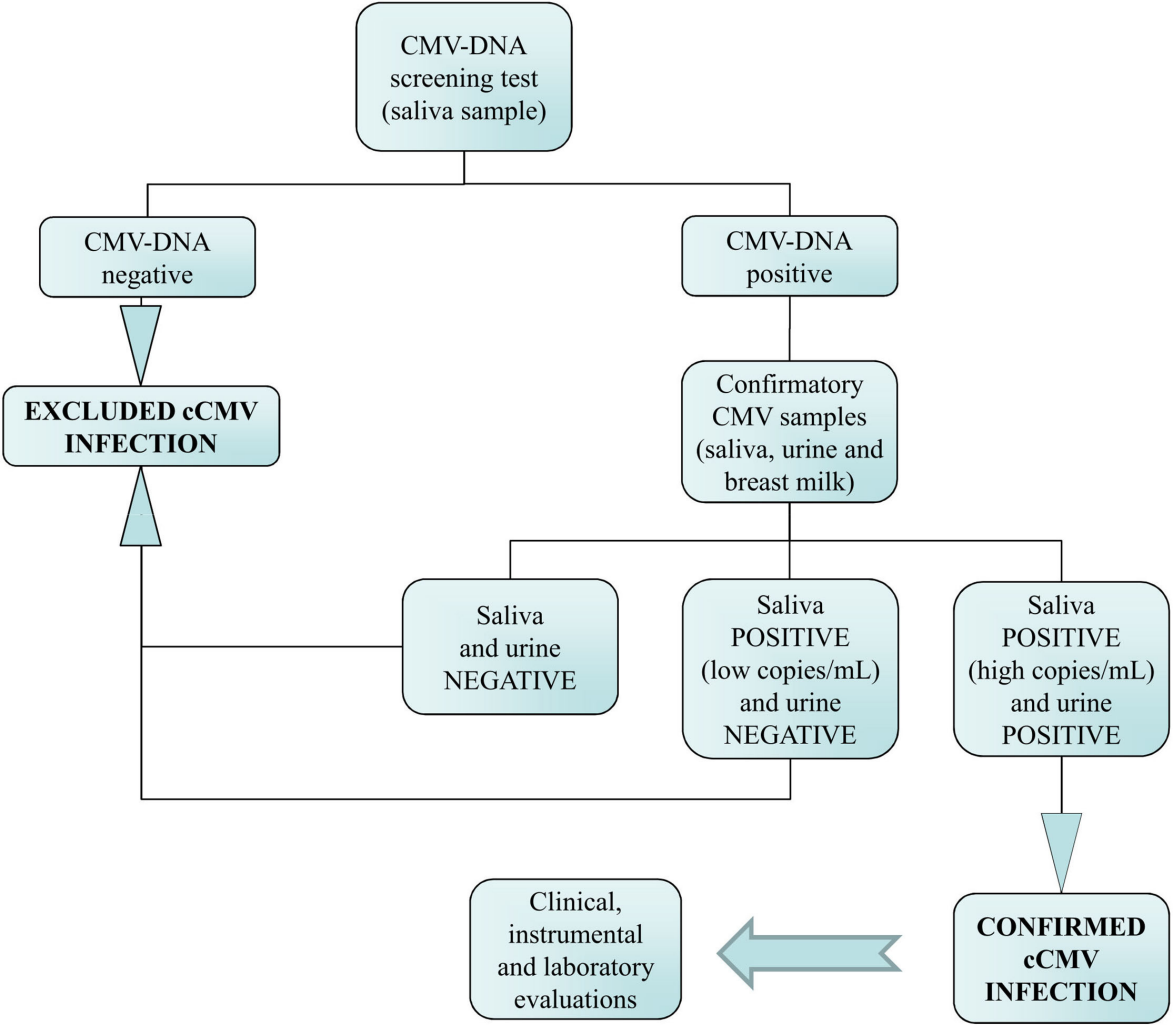
Flowchart of the neonatal congenital CMV (cCMV) screening and the algorithm used for the diagnosis of infection.

The flowchart of the cCMV screening and the algorithm used for the interpretation of the molecular results is summarized in Figure 1. Briefly, infants with CMV-DNA-positive screening results on a first salivary swab were recalled within 21 days of age for both a repeated saliva sample and a urine sample to confirm or exclude cCMV infection. Furthermore, in order to identify in the saliva screening samples a potential CMV-DNA contamination derived from breastfeeding, a breast milk sample was collected from mothers of these infants.

Infants diagnosed with cCMV infection underwent clinical, laboratory, and instrumental evaluations during the first month of life to define infection as symptomatic or asymptomatic and were followed up at least for the first 12 months of life (15).

Maternal CMV-serostatus before or during pregnancy was evaluated for all the neonates enrolled, i.e., values of anti-CMV immunoglobulin G (IgG) and anti-CMV immunoglobulin M (IgM) (positive/negative/equivocal) as well as anti-CMV IgG avidity indexes (low/moderate/high) were collected consulting patient's medical records. Serological data, routinely obtained at the three sites, i.e., using LIAISON^®^ CMV IgG, IgM, and IgG Avidity II assays (DiaSorin S.p.A., Saluggia, Italy), were interpreted according to the manufacturer's instruction. Maternal primary and non-primary infections were defined, as previously reported (4).

The study was approved by all three centers' Ethics Committee (i.e., Comitato Etico Indipendente di Area Vasta Emilia Centro, Comitato Etico Milano Area 3, Comitato Etico indipendente, and AOU Policlinico di Bari). Parents or guardians provided written consent prior to the inclusion of the infants into the study.

### Sample Collection and Storage

Saliva samples from neonates were collected by swabbing inside the mouth using a sterile flocked swab (FLOQSwabs^®^, Copan, Brescia, Italy); after collection, the swabs were immediately placed in the Universal Transport Medium (UTM^®^, Copan, Brescia, Italy).

Urine samples and breast milk samples were collected in a sterile container without a medium transport.

In the three centers, samples were investigated by using a single-automated quantitative commercial PCR assay (refer to below) within 48 h after collection; in addition, samples were consistently handled in terms of collection, transport, and storage conditions in order to minimize the quantification variability due to the pre-analytical phase.

### Molecular Assay

Saliva, urine, and breast milk samples were extracted, amplified, and quantified on the ELITeInGenius platform (ELITechGroup Molecular Diagnostics, Turin, Italy), a fully automated sample-to-result PCR system.

DNA was extracted from saliva, urine, and breast milk (200 μl of each body fluid eluted in 100 μl of elution buffer) using the ELITeInGenius total nucleic acid extraction kit (ELITechGroup Molecular Diagnostics, Turin, Italy) specifications with all parameters pre-programmed. CMV-DNA was detected and quantified with the real-time PCR assay CMV ELITe MGB kit (ElitechGroup Molecular Diagnostics, Turin, Italy), according to the manufacturer's package insert. Extraction, amplification, detection, and fully automated PCR analyses were performed on the ELITeIngenius System (ELITechGroup Molecular Diagnostics, Turin, Italy) in accordance with the manufacturer's specifications with onboard automation.

The viral load was reported as number of IU (International Unit)/ml for all body fluids examined. In association with the ELITeIngenius platform, the lower and upper limits of the detection of PCR assay for saliva samples were 44 IU/ml (220 gEq/ml) and 10^6^ IU/ml (5 × 10^7^ gEq/ml), respectively, the lower and upper limits for urine sample were 151 IU/ml (216 gEq/ml) and 3.5 × 10^7^ IU/ml (5 × 10^7^ gEq/ml), respectively, and the lower and upper limits for breast milk sample were 250 gEq/ml and 2.5 × 10^7^gEq/ml, respectively.

### Statistical Analysis

The study population characteristics were summarized using absolute frequencies and percentages and mean (standard deviation) for categorical and continuous variables, respectively. Variables representing elapsed time were summarized using median and range. Viral loads were transformed from IU/ml into log_10_ IU/ml to reduce data skewness. Comparisons between independent subgroups of samples, specifically false-positive vs. true-positive samples, were performed using the Mann–Whitney U-test; viral loads detected in the same patients at screening and confirmation tests were compared using the Wilcoxon matched-pairs test.

Statistical significance was set at α = 0.05. G^*^Power version 3.1.9.2 was used for sample size estimation; Stata version 15.1, JASP version 0.16.0.0, and GraphPad Prism version 9 were used for other analyses.

## Results

### Neonates Enrolled

During the study period, a total of 3,151 newborns were enrolled. All infants were born at a gestational age above 34 weeks, had no significant perinatal complications, and were enrolled mainly in the first 72 h of life. A small proportion of them (2%) required ventilation after birth with a bag-mask or Neopuff. None of the enrolled neonates required further steps of resuscitation, i.e., chest compression, endotracheal intubation, or drugs. Notably, 84 out of the 3,235 (2.6%) parents approached for this study refused the CMV newborn screening.

The baseline characteristics of the newborns at the time of enrollment and the maternal serological results available before or during pregnancy are summarized in Table 1.

**Table 1:** Baseline characteristics of the study population at the time of enrollment.

**Sex, number (%)**
Female	1,536 (48.7)
Male	1,615 (51.3)
Age	2 (1.13)
Mean value in days (SD)
Time elapsed from the saliva sample collection and the last breastfeeding (data	
were available for 73.2% [*n* = 2,307] of the study population)	
Median value in h (range)	2.0 (0–15.1*)
Number (%) of mothers who received tests for CMV-specific antibodies before	
or during pregnancy and classified on the base of serological results (data	
were available for 91.6% [*n* = 2,887] of the study population)	
Anti-CMV IgG positive and IgM negative**	2,014 (69.8)
Anti-CMV IgG negative and IgM negative	821 (28.4)
Anti-CMV IgG positive and IgM positive	52 (1.8)

^*^Breastfeeding with only breast milk was considered, and artificial feeding was not included. ^**^Only this CMV-serostatus was considered if performed before pregnancy.

Regarding maternal CMV immunity, a total of 2,014 (69.8%) mothers were CMV IgG positive and CMV IgM negative, 821 (28.4%) mothers were CMV IgG and IgM negative, and 52 (1.8%) mothers were CMV IgG and IgM positive. Maternal serological status was significantly different in the three study centers (χ^2^ test, *p* < 0.001), i.e., Bologna showed a higher proportion of women with CMV IgG- and IgM-negative results than Legnano-Magenta and Bari (33.2 vs. 25.7 vs. 22.2%, respectively) as well as a higher proportion of women with CMV IgG- and IgM-positive results than Legnano-Magenta and Bari (3.4 vs. 0.7 vs. 0.3%, respectively).

### Congenital CMV Infection Screening Results

The first saliva specimen was collected from the infants at a mean age of 2.25 days (SD, 1.13). Among the 3,151 investigated screening specimens, 3,076 (97.6%) samples were found to be CMV-DNA negative, and the cCMV infection was excluded. The remaining 75 (2.3%) saliva samples resulted positive for the detection of CMV-DNA (median viral load, 1.87 log_10_ IU/ml; range, 1.87–7.17 log_10_ IU/ml) and underwent confirmatory testing.

**Figure 2:**
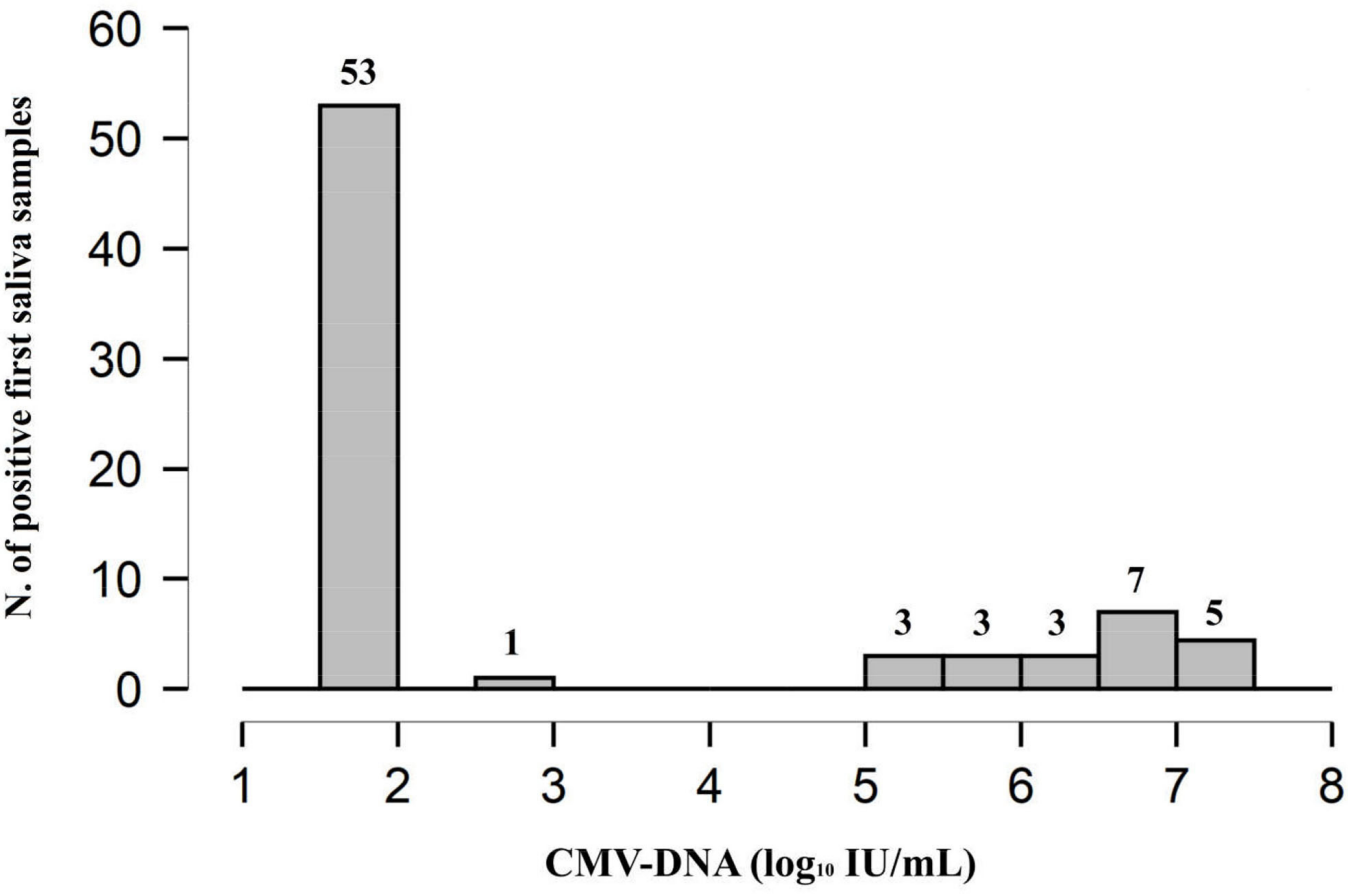
Distribution plot of CMV-DNA load in the 75 (2.3%) positive saliva screening samples. The remaining 3,076 (97.6%) samples were found to be CMV-DNA negative at screening.

The distribution of the CMV-DNA load in the saliva samples suggested that the 75 infants with positive results could be divided into 3 groups, namely, very low viral load group (i.e., viral load < 1.87 log_10_ IU/ml) consisting of 53 (70.7%) infants, low viral load group consisting of 1 (1.3%) infant with a CMV-DNA value of 2.59 log_10_ IU/ml, and high viral load group (i.e., viral load of at least 5.03 log_10_ IU/ml) consisting of the remaining 21 (28.0%) infants (Figure 2).

### Congenital CMV Infection Confirmatory Results

**Figure 3:**
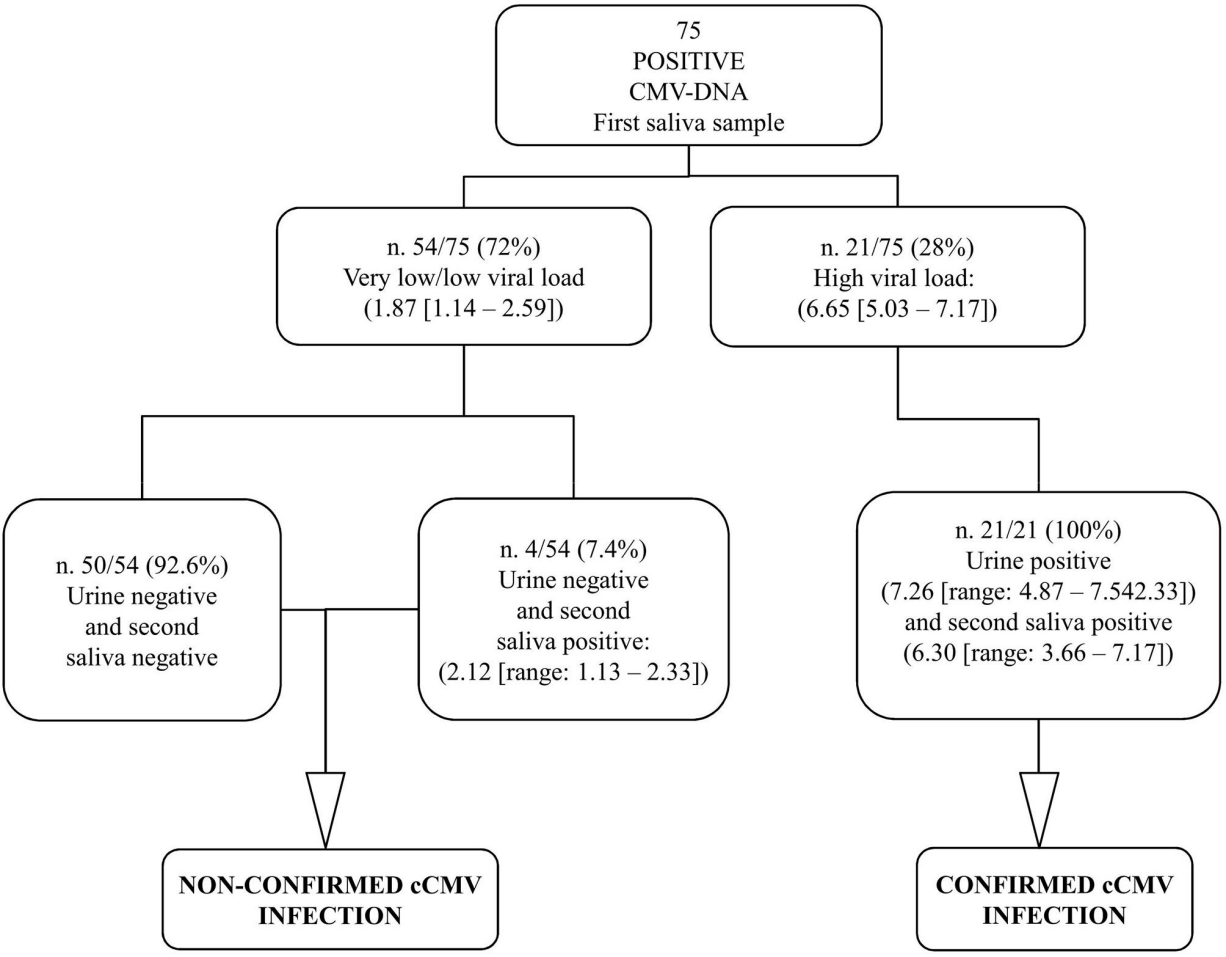
Graphical representation of the results obtained by confirmatory testing. CMV-DNA loads were expressed as log_10_ IU/ml (median value, [range]).

According to protocol, in order to diagnose cCMV infection, a second saliva sample and a urine sample were collected within 21 days of age (median 3, range, 1–18) from all the 75 positive infants; the mean age of the infants at the time of collection was 7.2 days (SD, 4.8). The results obtained by investigating the samples for confirmatory diagnosis are reported in Figure 3.

The 53 CMV-positive infants with very low viral load along with the single infant with low viral load at the screening had a CMV-negative urine sample and, therefore, were not confirmed to be congenitally CMV-infected; these were identified as false-positive first saliva samples. The repeated saliva sample was CMV-DNA negative in 50 (92.6%; 50/54) patients, including the unique patient with low viral load in the first saliva sample; the remaining four samples (7.4%; 4/54) resulted low CMV-DNA positive. All the 21 CMV-positive infants with high viral load from screening testing were confirmed to have cCMV infection since high CMV-DNA levels were detected in the urine samples; these first saliva samples were identified as true positive. Of note, among these 21 infants, all the repeated saliva samples resulted CMV-DNA positive with high viral load (Figure 3).

**Figure 4:**
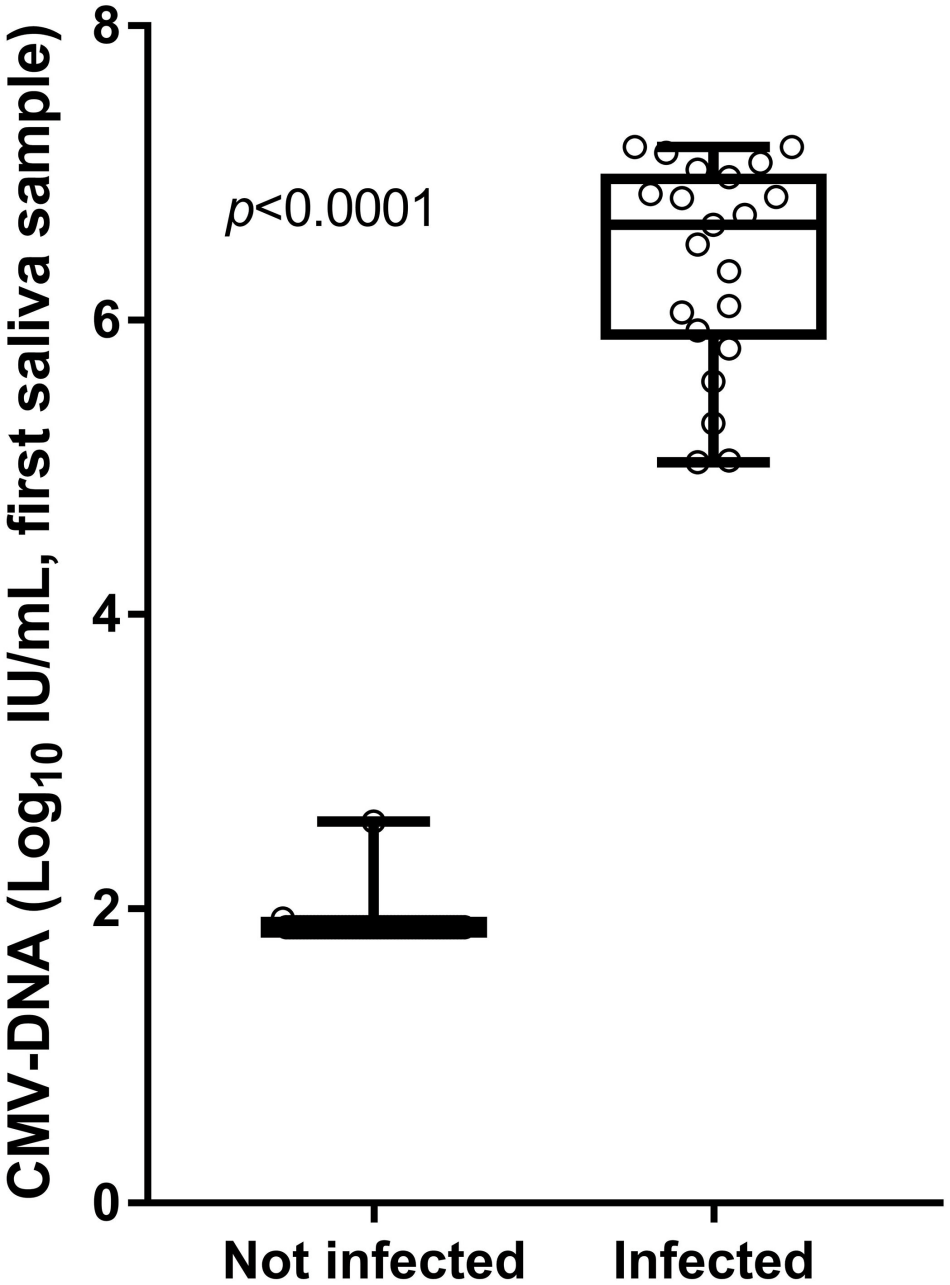
Viral loads detected in screening saliva samples from infants with and without cCMV infection.

The CMV-DNA loads detected in the screening saliva samples of the 21 confirmed congenitally CMV-infected infants (true positive) were higher than those detected in the 54 infants for whom the cCMV infection was excluded by confirmatory testing (false positive). The median CMV-DNA values were, respectively, 6.65 vs. 1.87 log_10_ IU/ml (Mann–Whitney U-test: *p* < 0.001; Figure 4).

Finally, the viral load of the 21 CMV-DNA saliva samples that resulted positive on both screening and confirmation testing was very similar at the screening and confirmation tests (median value, 6.33 vs. 6.29 log_10_ IU/ml; Wilcoxon matched-pairs test: *p* = 0.373).

### Potential CMV-DNA Contamination Results

The time elapsed from the last breastfeeding and the collection of the saliva samples that resulted false positive (with very low/low CMV-DNA levels) at the time of screening was the same as that observed in the saliva samples that resulted CMV-DNA negative at this time point (i.e., median time in h, 2; Mann–Whitney U-test: *p* = 0.527); data were available for 64.8 and 73.5% of cases (35/54 samples and 2,262/3,076 samples, respectively).

By investigating the 39 (72.2%) available breast milk samples collected from the 54 mother–infant pairs with false-positive saliva results at the time of screening, 20 (51.3%) breast milk samples resulted CMV-DNA negative, and 19 (48.7%) samples resulted CMV-DNA positive (median viral load, 3.23 gEq/ml; range, 2.39–5.96 gEq/ml).

### Characteristics of Congenital and Maternal CMV Infection

The overall incidence of cCMV infection in the study population was 0.66% (21/3,151 infants). In particular, in Bologna, a higher incidence (1.08%) than in Legnano-Magenta (0.36%) and in Bari (0.24%) centers was observed (Fisher's exact test: *p* = 0.049).

Among the 21 congenitally infected infants, clinical and instrumental findings were consistent with cCMV infection for 2 (9.5%) infants who were classified as symptomatic at birth and underwent valganciclovir (VGCV) treatment for 6 months; the remaining 19 (90.5%) infants were asymptomatic. Among the 19 infants, 1 (5.3%) infant developed SNHL at 5 months of age and the remaining 18 (94.7%) infants remained asymptomatic during the follow-up period (median time, 365 [range, 365–429] days). Overall, the incidence of symptomatic cCMV infection was equal to 14.3% (three neonates).

The clinical characteristics of the 21 cCMV infections along with the type and timing of maternal CMV infections are reported in Table 2. Among the 21 cCMV-infected newborns' mothers, 9 (42.9%) mothers were CMV IgG and IgM positive, 7 (33.3%) mothers were CMV IgG positive and CMV IgM negative, and 2 (9.5%) mothers were CMV IgG- and IgM-negative (in both cases, a last serological testing was carried out at 27 weeks of gestation). The remaining 3 (14.3%) mothers did not receive any serological test for CMV before or during pregnancy. Available serological data allowed us to define maternal CMV infection as primary in 10 (47.6%) newborns; 50% of these were observed in the third trimester. Seven (33.3%) newborns were born from non-primary maternal infection and in 6 cases (85.7%), it was not possible to define the onset of maternal CMV infection during the gestation. In the remaining cases (4.8%), given that only a result of CMV IgG positive and IgM negative in 22 weeks of gestation was available, it was not possible to define the type of maternal CMV infection.

**Table 2:** Characteristics of the 21 neonates with cCMV infection: symptoms, antiviral therapy, and maternal CMV infection.

**N. of**	**Symptoms**	**Symptoms during**	**Clinical**	**Antiviral**	**Maternal**		**Weeks of gestation at**	**Type of**	**Onset of maternal**
**infants**	**at birth**	**the follow-up**	**symptoms**	**therapy**	**CMV**		**the moment of execution of**	**maternal**	**CMV infection**
		**period**		**administration**	**serostatus**		**the first serological tests**	**infection**	**Trimester of pregnancy**
					**Anti-CMV**	**Anti-CMV**			
					**IgG/ IgM**	**IgG avidity index**			
1	**YES**	NO (symptoms	Bilateral SNHL profound	YES*	+/+	Low	10 weeks	Primary	I trimester
		at birth persist)	on the right moderate
			on the left
1	NO	**YES** At 5	Moderate unilateral	NO	+/+	Low	11 weeks	Primary	I trimester
		months after birth	SNHL
1	NO	NO	NO	NO	+/+	NM	24 weeks	Primary	II trimester
2	NO	NO	NO	NO	+/+	Low/moderate	26 weeks both	Primary	II trimester
1	NO	NO	NO	NO	+/+	NM	31 weeks	Primary	III trimester
2	NO	NO	NO	NO	+/+	Low	35 weeks both	Primary	III trimester
2	NO	NO	NO	NO	–/–	/	27 weeks both	Primary	III trimester
1	NO	NO	NO	NO	+/+	High	10 weeks	Non-primary	I trimester
6	NO	NO	NO	NO	+/–	NA	range, 9 – 25weeks	Non-primary	Undefined
1	NO	NO	NO	NO	+/–	NA	22 weeks	Active^∧^	Undefined
								infection not defined	
2	NO	NO	NO	NO	NA
1	**YES**	NO (symptoms	Profound unilateral	YES*	NA
		at birth persist)	SNHL CNS
			involvement

+, positive; –, negative; SNHL, sensorineural hearing loss; CNS, central nervous system; NM, not measurable due to low anti-CMV IgG levels; NA, not available. *Standard dose, oral valganciclovir 16 mg/kg twice daily/6 months; ^∧^Maternal positive CMV-DNAemia.

Finally, the proportion of maternal CMV-specific IgG- and IgM-positive results, evaluated separately, was compared between the neonates with and without cCMV infection; a statistical significance was observed only for the IgM maternal positivity (Table 3).

**Table 3:** CMV immunity assessed before or during pregnancy of the study participants' mothers.

**Maternal CMV-specific antibodies**	***N*. 2,869 NO cCMV infection *n* (%)**	***N*. 18 YES cCMV infection *n* (%)**	*p*-value*
IgG positive	2,055 (71.5)	16 (88.9)	0.121
IgM positive	43 (1.5)	9 (50.0)	**<0.001**

Data were available for 85.7% (18/21) and 67% (2,869/3,130) of the infants with and without cCMV infection, respectively. *Fisher's exact test.

## Discussion

The results obtained by carrying out a universal screening for cCMV infection of all live-born neonates in three different centers in Italy by using the saliva PCR assay, as well as the therapeutic interventions and the clinical outcome of the CMV-infected neonates were described; the characteristics of the maternal CMV infection were also evaluated.

A very high (97.4%) parental acceptability of newborn screening was observed. In the study population, the overall incidence of cCMV infection was equal to 0.66%, in line with literature data (12–14). Of note, a different incidence of cCMV infection among the three study centers was found, with the highest percentage (1.08%) observed in the Bologna one, reflecting the different distribution of maternal CMV immunity among the three centers (16). Particularly, CMV IgM-positive results were associated with a higher proportion of congenitally CMV-infected neonates than uninfected (*P* < 0.001), and the highest percentage of mothers with a positive value of anti-CMV IgM was observed in the Bologna center. This finding is likely because this center is a large national referral center for diagnosis and counseling of CMV infection during pregnancy as well as for the clinical management of congenitally infected newborns. Overall, maternal CMV immunity was known in a very high percentage of cases (91.6%).

At the time of screening, cCMV infection was excluded in almost all cases (97.62%) by means of PCR-negative first saliva sample result. The remaining 2.38% of cases underwent confirmatory investigations. In particular, all (100%) the neonates with a high viral load (at least 5.03 log_10_ IU/ml) in screening saliva samples were confirmed to be CMV congenitally infected by means of high levels of CMV-DNA detected in urine samples, as well as in the repeated saliva samples. In contrast, in all the neonates with low and very low viral load (< 2.59 log_10_ IU/ml) in screening saliva sample, cCMV infection was excluded by means of PCR-negative urine sample. These findings confirm those suggested by other authors (16, 17) that the evaluation of viral load measured in the first saliva sample could be helpful to discriminate between true-positive and false-positive results. False-positive results were associated with low viral loads, whereas high DNA levels were found only in true-positive samples (*P* < 0.001). Specifically, by comparing the highest value of viral load found at the time of screening in the false-positive saliva samples (2.59 log_10_ IU/ml) with the lowest one found in the true-positive saliva samples (5.03 log_10_ IU/ml), a difference in CMV-DNA load equal to 2.44 log_10_ IU/ml was observed. Considering these results, it is reasonable to suggest that a saliva viral load of < 2.59 log_10_ IU/ml may be indicative of a newborn without cCMV infection and is most likely a result of contamination; however, low viral load in neonatal saliva could potentially be observed in case of intrauterine CMV transmission at the end of the third trimester, as previously reported in neonatal urine by Exler et al. (18). In 7.4% of the infants with a CMV-DNA very low/low positive first saliva sample, the repeated saliva samples also resulted CMV-DNA positive with very low viral load (i.e., the maximum value of CMV-DNA detected was equal to 2.34 log_10_ IU/ml). These findings in agreement with recent literature (17–20) showed that a definitive diagnosis of cCMV, avoiding unnecessary tests and waste of resources, is to be confirmed by investigating urine sample that remains the gold standard for the diagnosis of cCMV infection. However, the collection of urine samples can be difficult and time-consuming compared to the saliva collection (8, 9). Therefore, saliva sampling in newborn screening programs is easier and more practical. In this study, in line with others (16, 20, 21), a low overall percentage (1.7%) of false-positive saliva screening samples was observed, confirming the suitability of this testing methodology. It is known that a potential CMV-DNA contamination may result from the breastfeeding (9, 22, 23). In this regard, the same median interval from the last breastfeeding and the screening salivary swab collection was observed in the false-positive samples and the true-negative samples, and CMV-DNA was detected in only almost half of the breast milk samples of mother–infant pairs with a false-positive result, suggesting that contamination from breast milk may not be the unique explanation in our study population. Of note, a large number of false-positive results at the time of screening were observed in the early period of the study (data not shown) and this led to setting up a strict operational procedure, consistent between all the centers, to avoid any potential contamination during specimen collection.

In line with literature data (15, 24), most infected neonates were asymptomatic at birth and during follow-up. Two (9.5%) infants showed symptoms consistent with cCMV infection at birth and received a 6-month course of antiviral therapy, i.e., one case with severe disease (central nervous system involvement and profound unilateral SNHL) and one case with isolated bilateral SNHL (moderate on one side and profound on the other side). These symptomatic newborns at birth would have also been identified by a CMV-targeted screening, since both of them would have failed the universal newborn hearing screening. One asymptomatic neonate developed moderate unilateral SNHL at 5 months of age. Considering both early- and late-onset symptoms, the percentage of symptomatic CMV infection in the study population was 14.3%.

Of note, 10 out of the 21 (47.6%) congenitally infected infants would not have undergone early virological, instrumental, and clinical evaluation for cCMV infection. Of note, in a phase II international, multi-center, double-blind, placebo-controlled trial of 6 weeks of oral VGCV or 6 weeks of placebo for infants/toddlers with cCMV infection and hearing loss for 1 month through 3 years of age that aimed to assess whether the 6-week course of oral VGCV could stabilize the hearing function (ClinicalTrials.gov Identifier: NCT01649869), no difference in the placebo and treatment groups in terms of the hearing outcome was observed, suggesting that a delayed diagnosis of cCMV after the neonatal period is a missed opportunity for antiviral treatment, which has been demonstrated to confer some benefits in terms of hearing function and neurodevelopmental outcome only if started in the first month of life (25). Due to the increase in the workload of health workers and the expense of molecular testing for CMV of neonatal screening programs, national studies are needed to evaluate the feasibility and cost-effectiveness of an Italian universal newborn CMV screening. By analyzing the outcome of the neonatal infection in relation to the type of CMV maternal infection (the information was available in 80.9% of cases), similar to Faure-Bardon and colleagues (26), it was observed that the two cases of primary maternal infection in the first trimester were both associated with cCMV symptomatic infection; the remaining symptomatic neonate was born to a mother with no CMV-checkups in pregnancy. cCMV infections were a result of non-primary maternal CMV infection in approximately one-quarter of the defined cases and were all asymptomatic. Whether the risk of symptomatic cCMV infection, especially that resulting in hearing loss, differs following primary or non-primary maternal infection has been a matter of debate in the past years. Nevertheless, recent evidence suggests that there is no difference in the risk of hearing loss according to the type of maternal CMV infection (16, 27–29).

The strengths of this study were the large prospective sample size evaluated, the adoption of a single-automated molecular method and a single experimental approach both common to all the study centers, and the timing of the collection of the cCMV infection confirmatory samples that occurred in all cases and for all types of samples inside the timeframe recommended of 21 days of age (11) as well as the maternal CMV immunity that was known in almost all cases of study participants and the availability of all the clinical information about the congenitally infected neonates identified during the CMV screening. The limitations are that the type of maternal infection was unknown in 19.0% of the cCMV infections, including one symptomatic case, and the number of cCMV-infected newborns was too small for evaluating the risk of hearing loss according to the type of maternal CMV infection.

In conclusion, in our setting of neonatal CMV screening, the percentage of false-positive results in saliva samples was low. In particular, low positivity for CMV-DNA in saliva samples was associated with false-positive results. This finding could be communicated to parents by limiting their stress and anxiety while waiting for the confirmatory diagnostics. Screening of cCMV infection by saliva molecular testing and subsequent confirmation with the search of CMV-DNA in a urine sample, rather than in a repeated saliva sample, proved to be the gold standard strategy. Finally, despite universal neonatal screening for CMV is not currently recommended by any public health body (4) and infected neonates are, therefore, identified only because of suspected maternal infection during pregnancy or symptoms and signs associated with cCMV at birth, our findings confirmed literature data reporting that without neonatal screening, some infected infants at risk to develop neurological sequelae may not be recognized earlier.

## Data Availability Statement

The original contributions presented in the study are included in the article/supplementary materials, further inquiries can be directed to the corresponding author/s.

## Ethics Statement

The studies involving human participants were reviewed and approved by Comitato Etico Indipendente di Area Vasta Emilia Centro, Comitato Etico Milano Area 3, Comitato Etico indipendente, AOU Policlinico di Bari. Written informed consent to participate in this study was provided by the participants' legal guardian/next of kin.

## Author Contributions

ACh collected and analyzed the data and wrote, reviewed, and edited the manuscript. CP, MS, and ACa performed the analyses and collected and analyzed the data. GT participated in analyzing the data and contributed to writing the first draft of some sections of the manuscript. EB, LG, and MD participated in collecting and analyzing the data. DG performed formal analysis. FB and MM performed analyses and participated in collecting results. CM, AR, LPo, MB, AP, LPa, MCapo, MCapr, and NL clinically managed the infants and provided related information. PC supervised the research and contributed to the data analysis. TL conceptualized and supervised the research, analyzed the data, and reviewed and edited the manuscript. All authors contributed to the article and approved the submitted version.

## Funding

This study was supported by ELITechGroup Molecular Diagnostics, Turin, Italy.

## Conflict of Interest

The authors declare that the research was conducted in the absence of any commercial or financial relationships that could be construed as a potential conflict.

## Publisher's Note

All claims expressed in this article are solely those of the authors and do not necessarily represent those of their affiliated organizations, or those of the publisher, the editors and the reviewers. Any product that may be evaluated in this article, or claim that may be made by its manufacturer, is not guaranteed or endorsed by the publisher.
